# A Novel Intelligent Fault Diagnosis Method for Self-Priming Centrifugal Pumps

**DOI:** 10.3390/e25111501

**Published:** 2023-10-30

**Authors:** Bo Zhang, Zhenya Wang, Ligang Yao, Biaolin Luo

**Affiliations:** 1 School of Mechanical Engineering and Automation, Fuzhou University, Fuzhou 350108, China; 15551251180@163.com (B.Z.); 15759212532@163.com (B.L.); 2 Department of Mechanical Engineering, Tsinghua University, Beijing 100084, China

**Keywords:** self-priming centrifugal pump, fault diagnosis, fluctuation dispersion entropy, manifold mapping, support vector machine

## Abstract

The real-time diagnostic monitoring of self-priming centrifugal pumps is essential to ensure their safe operation. Nevertheless, owing to the intricate structure and complex operational conditions inherent in such pumps, existing fault diagnosis methods encounter challenges in effectively extracting crucial fault feature information and accurately identifying fault types. Consequently, this paper introduces an intelligent fault diagnosis method tailored for self-priming centrifugal pumps. The approach amalgamates refined time-shift multiscale fluctuation dispersion entropy, cosine pairwise-constrained supervised manifold mapping, and adaptive chaotic Aquila optimization support vector machine techniques. To begin with, refined time-shift multiscale fluctuation dispersion entropy is employed to extract fault-related features, adeptly mitigating concerns related to entropy domain deviations and instability. Subsequently, the application of cosine pairwise-constrained supervised manifold mapping serves to reduce the dimensionality of the extracted fault features, thereby bolstering the efficiency and precision of the ensuing identification process. Ultimately, the utilization of an adaptive chaotic Aquila optimization support vector machine facilitates intelligent fault classification, leading to enhanced accuracy in fault identification. The experimental findings unequivocally affirm the efficacy of the proposed method in accurately discerning among various fault types in self-priming centrifugal pumps, achieving an exceptional recognition rate of 100%. Moreover, it is noteworthy that the average correct recognition rate achieved by the proposed method surpasses that of five existing intelligent fault diagnosis techniques by a significant margin, registering a notable increase of 15.97%.

## 1. Introduction

Self-priming centrifugal pumps, as indispensable key equipment in the industrial field, have recently played an important role in energy conversion and drive actuators [1]. Nevertheless, the self-priming centrifugal pump is susceptible to failures or significant accidents caused by component damage due to prolonged operation and harsh working conditions. Therefore, accurate and timely diagnosis of self-priming centrifugal pump faults is significant for improving equipment reliability, ensuring production continuity, and reducing maintenance costs. However, the operating state of self-priming centrifugal pumps is usually characterized by variability and complexity. Traditional fault diagnosis methods include state estimation, time-frequency analysis, statistical methods, etc. These methods depend on experience and equipment, are costly in time and manpower, and are highly restrictive.

The continuous advancement of machine learning and artificial intelligence technologies has led to the widespread application of data-driven techniques across various domains, including manufacturing, healthcare, transportation, finance, and energy. These innovations are driving progress and development in diverse industries. For instance, in the realm of robotics, Peng et al. introduced the Funabot-Suit, a biologically inspired garment propelled by McKibben muscles, enabling natural proprioceptive perception [2]. Meanwhile, Mao et al. devised a predictive modeling approach for flexible electro-hydrodynamic pumps, leveraging soft computing techniques [3]. Moreover, data-driven methodologies have found substantial utility in the domain of rotating machinery failure analysis. Zhou et al. put forth a deep convolutional generative adversarial network to achieve precise diagnostics with limited labeled data, exemplifying the capabilities of these techniques [4]. Han et al. introduced an innovative framework tailored for addressing the challenge of transfer diagnosis with sparse target data. This approach not only reduces distribution disparities but also mitigates undesirable transitions [5]. In a similar vein, Wu et al. proposed an adaptive deep transfer learning method for bearing fault diagnosis [6].

Data-driven fault diagnosis methods provide significant advantages over traditional approaches, including increased levels of automation, enhanced accuracy, and multidimensional analysis capabilities. This results in more timely and precise fault diagnosis outcomes, ultimately contributing to improved pump reliability and operational efficiency. Notably, this method comprises two pivotal steps: feature extraction and pattern recognition [7,8].

To commence, it is imperative to extract pertinent features from the vibration signal originating from the self-priming centrifugal pump; however, it is observed that the vibration signal of the self-priming centrifugal pump typically demonstrates characteristics of non-stationarity, nonlinearity, and complexity. Conventional feature extraction methods operating in time, frequency, or time–frequency domains are susceptible to a range of challenges. These issues encompass information loss, fluctuations in signals, subjective and unstable artificial feature selection, noise interference, and reliance on domain knowledge or experiential input [9]. Consequently, entropy-based methods have been developed in line with the advancements in nonlinear dynamics technology. These methods encompass symbolic dynamic entropy, Shannon entropy, multiscale entropy, sample entropy, permutation entropy, multiscale dispersion entropy (MDE), and multiscale fluctuation dispersion entropy (MFDE) [10,11,12,13].

Within the context of the aforementioned entropy theories, it is worth noting that MFDE can effectively gauge the regularity of the time series, thereby enabling the detection of subtle variations within the vibration signals. MFDE is advantageous for its rapid computational speed and robust resistance to noise. Nevertheless, it does exhibit certain limitations when applied to the extraction of fault features from faulty signals. Notably, as the scale factors increase, the coarse-grained sequence undergoes shortening, leading to substantial deviations in the larger factors [12]. Wang et al. proposed the refined time-shift multiscale fluctuation dispersion entropy (RTSMFDE), whose powerful feature extraction capability was validated by fault diagnosis experiments of a wind turbine [7]. The time-shift multiscale decomposition was used to replace the original mean multiscale decomposition based on the MFDE, preserving the important structure information of the signal and making the obtained entropy more accurate and stable. In addition, the entropy calculation was carried out using a refined method, i.e., the relative frequencies of fluctuation dispersion modes of all time-shifted coarse-granulated sequences under the scale factors were first averaged, and then the entropy was calculated, reducing the possibility of invalid entropy. Given the advantages of the RTSMFDE, this paper applies it to the fault feature extraction of self-priming centrifugal pumps.

Nonetheless, when the RTSMFDE feature set is directly utilized as the input for the pattern recognition classifier, there is a potential compromise in the classifier’s recognition accuracy; this arises due to the high dimensionality and the presence of information redundancy within the RTSMFDE feature set. Consequently, the implementation of a dimensionality reduction (DR) method becomes imperative to obtain low-dimensional and discriminating feature sets [14].

Traditional DR methods, such as linear discriminant analysis (LDA) and principal component analysis, are all linear methods incapable of handling nonlinear feature sets [15,16]. In contrast, manifold learning, which is a nonlinear DR method, is an effective approach for uncovering low-dimensional structures within high-dimensional spaces, offering a more suitable solution for DR applied to the data collected from hydraulic pumps [17,18]. Commonly used manifold learning methods include isometric mapping (Isomap), Laplacian eigenmaps (LE), locally linear embedding (LLE), and local tangent space alignment (LTSA) [19,20,21,22]. However, their application in reducing the dimensionality of the self-priming centrifugal pump fault feature set has some limitations. For instance, the above-mentioned methods are unsupervised DR approaches that fail to leverage the available sample label information fully; hence, the DR result is easily disturbed by noise points. The aforementioned methods use Euclidean distance to construct a neighborhood graph easily affected by dimension. In cases where certain outliers are treated as near neighbors, the Euclidean distance metric may fail to establish a meaningful relationship between isolated samples and their other close neighbors; this can lead to the disruption of the underlying neighborhood graph structure [23].

The recently proposed cosine pairwise-constrained supervised manifold mapping (CPCSMM) method aims to extract both local and global structural information from signal features, effectively reducing and visualizing high-dimensional data [7]. Due to its exceptional performance in the DR of fault features, this paper employs the CPCSMM method for reducing the dimensionality of a high-dimensional RTSMFDE feature set. Consequently, a low-dimensional and discerning fault feature set can be obtained.

The extracted feature set should be input into the classifier for recognition to achieve the intelligent diagnosis of a self-priming centrifugal pump. Common methods include the K-nearest neighbor, naive Bayes, artificial neural network, and support vector machine (SVM) [24]. However, the K-nearest neighbor requires excessive computation costs when dealing with high-dimensional and large-scale data. Unbalanced data distribution and inappropriate parameter settings will significantly affect the accuracy of K-nearest neighbor classification [25,26]. The naive Bayes method computes prior probabilities and operates under the assumption that the target attributes are conditionally independent of one another, which may not be entirely accurate when dealing with certain fault features in rotating machinery [27]. The complexity of artificial neural networks increases with the input data dimension and can easily overfit. This artificial neural network lacks strict theoretical support, while the black box model is poorly interpreted [28,29,30,31].

When juxtaposed with the previously mentioned methods, the SVM classifier delivers superior classification outcomes and holds notable advantages in managing scenarios involving limited sample sizes and nonlinear data. This is achieved by striking an optimal equilibrium between learning model accuracy and complexity. As a result, the SVM finds extensive application in the intelligent fault diagnosis of rotating machinery. However, the efficacy of the SVM is constrained by two core parameters of the classifier, namely the kernel parameter *g* and the penalty factor $c^{\prime }$. Specifically, parameter *g* regulates the complexity of the feature subspace distribution, while parameter $c^{\prime }$ gauges the proportion of misclassified samples and the complexity of the model [10]. To enhance the SVM’s generalization ability and accuracy, the adaptive chaotic Aquila optimization support vector machine (ACAO-SVM) classifier was adopted, employing an adaptive optimization strategy [10]. The ACAO-SVM was further implemented in recognizing faults of self-priming centrifugal pumps.

The main contributions of this paper can be summarized as follows:

(1) The introduction of a novel intelligent fault diagnosis method tailored for self-priming centrifugal pumps, which integrates the refined time-shift multiscale fluctuation dispersion entropy, cosine pairwise-constrained supervised manifold mapping, and adaptive chaotic Aquila optimization support vector machine;
(2) The practical application of the proposed intelligent fault diagnosis method within the context of analyzing a self-priming centrifugal pump case. This endeavor serves the purpose of validating the method’s effectiveness;
(3) A comprehensive comparative analysis involving the proposed fault diagnosis method, various feature extraction techniques, feature dimensionality reduction methods, and existing intelligent fault diagnosis approaches. This comparative assessment is aimed at substantiating the method’s superior performance.

The organization of the remaining sections of the paper is as follows: in Section 2, the theoretical basis and specific process of the proposed intelligent fault diagnosis method for self-priming centrifugal pumps are presented. Section 3 conducts a case study on a self-priming centrifugal pump and compares it with existing methods of feature extraction, feature dimensionality reduction, and fault diagnosis. Lastly, Section 4 summarizes the paper, and the key conclusions are drawn, emphasizing the significant contributions and implications of the proposed intelligent fault diagnosis method for self-priming centrifugal pumps.

## 2. Intelligent Fault Diagnosis Model for Self-Priming Centrifugal Pump

### 2.1. Proposed Intelligent Fault Diagnosis Model

**Figure 1:**
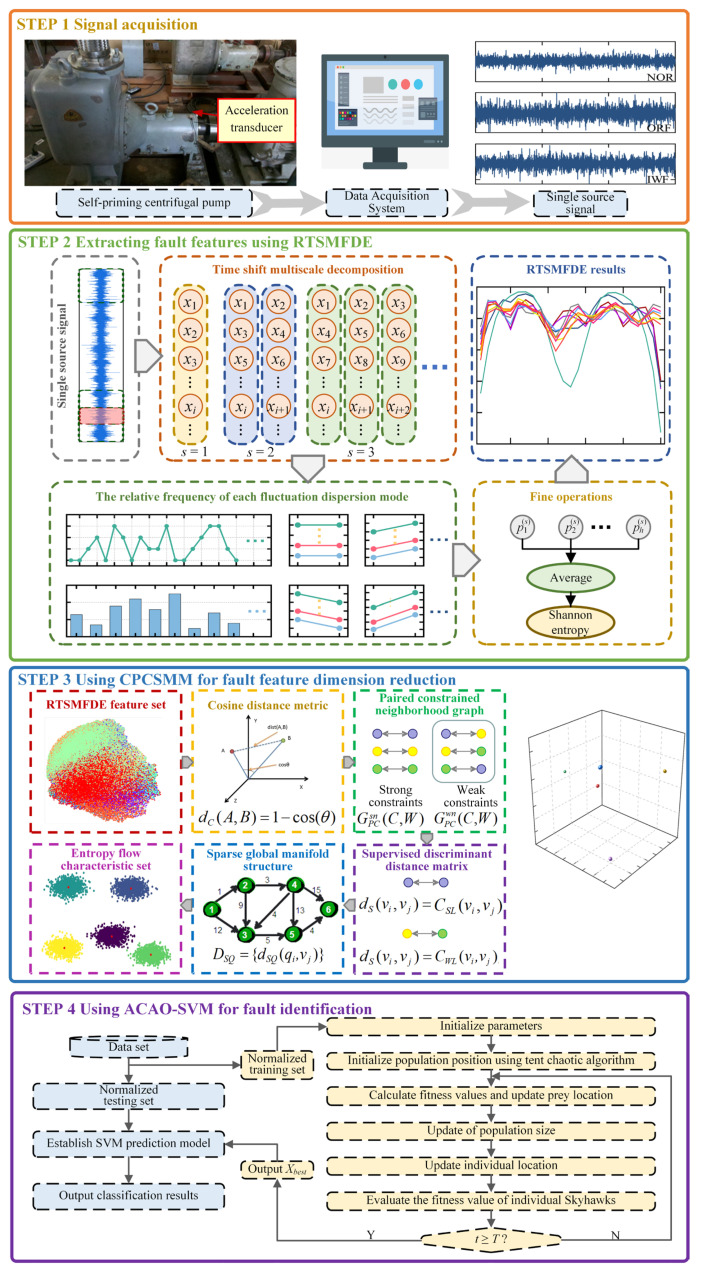
Intelligent fault diagnosis method.

Based on the RTSMFDE, CPCSMM, and ACAO-SVM, an intelligent fault diagnosis method for self-priming centrifugal pumps was devised. The methodology employed in this study involves a sequential process. Initially, the RTSMFDE method was employed for the extraction of fault-related information from the self-suction centrifugal pump. Subsequently, the CPCSMM method was applied to reduce the dimensionality of the RTSMFDE feature set, effectively isolating sensitive feature components. Lastly, the resulting low-dimensional feature set was fed into the ACAO-SVM classifier, facilitating intelligent fault recognition. Figure 1 illustrates the process, while the specific steps are detailed as follows:

(1) Signal acquisition. A single sensor obtains the self-priming centrifugal pump signal under different operating conditions;
(2) Feature extraction. The RTSMFDE extracts the entropy features of each group of signal samples and constructs the fault feature vector in the entropy domain;
(3) Dimensionality reduction. The dimensionality of the extracted high-dimensional fault feature set from the RTSMFDE is reduced by the CPCSMM method, resulting in an entropy-manifold feature set that exhibits a high degree of fault differentiation;
(4) Fault identification. The training set is constructed by randomly selecting entropy-manifold feature vectors from the samples. Conversely, the remaining samples’ entropy-manifold feature vectors are considered as the test set. Both the training and test sets undergo normalization. The normalized training set is used to construct the predictive model. Subsequently, the normalized test set is fed into the predictive model for intelligent fault diagnosis self-priming centrifugal pumps.

### 2.2. Refined Time-Shift Multiscale Fluctuation Dispersion Entropy

The signal was set as $X=\{x_{i}\;,i=1,\;\;\;2,\ldots ,N\}$, and the RTSMFDE process was as follows.

(1) Under the scale factor *s*, *h* time-shift multiscale decomposition sequences *X* are constructed:

(1) $$y_{h}^{\left(s\right)}=\{x_{h},\;x_{h+s},\;\ldots ,x_{h+[(N-h)/s]\;s}\},\;1\leq h\leq s$$

where the time-shift multiscale decomposition sequence is converted into the original signal when *s* = 1 and [(N−h)/s] is the nearest integer, which is less than (N−h)/s.

(2) The normal cumulative distribution function is used to decompose the time-shift multiscale subsequences mapping $y_{h}^{\left(s\right)}$ to $Y_{h}^{\left(s\right)}=\{{y^{\prime }}_{h,j}^{\left(s\right)},j=1,2,\ldots ,N/s\}$:

(2) $${y^{\prime }}_{h,j}^{\left(s\right)}=\frac{1}{\sigma \sqrt{2\pi }}\int_{-\infty }^{y_{h}^{\left(s\right)}}e^{\frac{-{\left(t-u\right)}^{2}}{2\sigma^{2}}}dt$$

where ${y^{\prime }}_{h,j}^{\left(s\right)}\in (0,\;\;\;1)$; σ and u represent the standard deviation and mean of $y_{h}^{\left(s\right)}$, respectively.

(3) $Y_{h}^{\left(s\right)}$ is mapped to an integer index from 1 to *c* using a linear transformation $z_{h}^{\left(c\right)}=\{z_{h,j}^{\left(c\right)}\}$:

(3) $$z_{h,j}^{\left(c\right)}=R(c\cdot {y^{\prime }}_{h,j}^{\left(s\right)}+0.5)$$

where R(⋅) represents the rounding function; *c* stands for the category. Since step (2) uses the normal cumulative distribution function, the mapping process can still be considered nonlinear.

(4) The reconstructed sequence $z_{h,i}^{\left(m,c\right)}$ is obtained using phase space reconstruction:

(4) $$z_{h,\;\;i}^{\left(m,\;\;c\right)}=\{z_{h,i}^{\left(c\right)},\;\;z_{h,i+t}^{\left(c\right)},\ldots ,\;\;z_{h,i+(m-1)\;\;t}^{\left(c\right)}\},\;i=1,\;\;2,\ldots ,\;(N/s)-(m-1)\;\;t$$

where *t* represents delay and *m* represents the embedding dimension.

(5) Fluctuation dispersion analysis for the reconstructed sequence:

(5) $$\begin{array}{l} {z^{\prime }}_{h,\;\;i}^{\left(m,\;\;c\right)}=\{z_{h,\;i+t}^{\left(c\right)}-z_{h,\;i}^{\left(c\right)},\;\;\;z_{h,\;i+2t}^{\left(c\right)}-z_{h,\;i+t}^{\left(c\right)},\ldots ,z_{h,\;i+(m-1)t}^{\left(c\right)}-z_{h,\;i+(m-2)t}^{\left(c\right)}\},\\ \;\;\;i=1,2,\ldots ,(N/s)-(m-1)t \end{array}$$

where the fluctuation dispersion mode of each sequence ${z^{\prime }}_{h,\;\;i}^{\left(m,\;\;c\right)}$ is defined as ${\pi^{\prime }}_{{g^{\prime \prime }}_{1}{g^{\prime \prime }}_{2}\ldots {g^{\prime \prime }}_{m-1}}$, $z_{h,i+t}^{\left(c\right)}-z_{h,i}^{\left(c\right)}={g^{\prime \prime }}_{1}$, $z_{h,i+t}^{\left(c\right)}={g^{\prime \prime }}_{2}$,..., $z_{h,i+(m-1)t}^{\left(c\right)}-z_{h,i+(m-2)t}^{\left(c\right)}={g^{\prime \prime }}_{m-1}$. In addition, the number of potential fluctuation dispersion modes assigned to the sequence ${z^{\prime }}_{h,\;\;i}^{\left(m,\;\;c\right)}$ is ${\left(2c-1\right)}^{m-1}$.

(6) The relative frequency $p_{h}^{\left(s\right)}({\pi^{\prime }}_{{g^{\prime \prime }}_{1}{g^{\prime \prime }}_{2}\ldots {g^{\prime \prime }}_{m-1}})$ of each fluctuation dispersion mode is calculated as follows:

(6) $$\begin{array}{l} p_{h}^{\left(s\right)}({\pi^{\prime }}_{{g^{\prime \prime }}_{1}{g^{\prime \prime }}_{2}\ldots g_{m-1}})=\frac{1}{(N/s)-(m-1)\;\;t}\mathrm{Number}\{i|i\leq (N/s)-\;(m-1)t,\;\;\\ \;{z^{\prime }}_{h,\;\;i}^{\left(m,\;\;c\right)}\;\;\;\mathrm{has}\;\;\mathrm{type}\;\;{\pi^{\prime }}_{{g^{\prime \prime }}_{1}{g^{\prime \prime }}_{2}\ldots g_{m-1}}\} \end{array}$$

(7) The average relative frequency ${\bar{p}}_{h}^{\left(s\right)}({\pi^{\prime }}_{{g^{\prime \prime }}_{1}{g^{\prime \prime }}_{2}\ldots {g^{\prime \prime }}_{m-1}})$ of multiple time-shift multiscale decomposition sequences with scale factor *s* is calculated in a refined way:

(7) $${\bar{p}}_{h}^{\left(s\right)}({\pi^{\prime }}_{{g^{\prime \prime }}_{1}{g^{\prime \prime }}_{2}\ldots {g^{\prime \prime }}_{m-1}})=\frac{1}{s}\sum_{h=1}^{s}p_{h}^{\left(s\right)}({\pi^{\prime }}_{{g^{\prime \prime }}_{1}{g^{\prime \prime }}_{2}\ldots {g^{\prime \prime }}_{m-1}})$$

(8) The RTSMFDE can be expressed as follows:

(8) $$\mathrm{RTSMFDE}\;(X,c,m,\;\;\;t,s)=-\sum_{\pi =1}^{{\left(2c-1\right)}^{m-1}}{\bar{p}}_{h}^{\left(s\right)}({\pi^{\prime }}_{{g^{\prime \prime }}_{1}{g^{\prime \prime }}_{2}\ldots {g^{\prime \prime }}_{m-1}})\cdot \ln{\bar{p}}_{h}^{\left(s\right)}({\pi^{\prime }}_{{g^{\prime \prime }}_{1}{g^{\prime \prime }}_{2}\ldots {g^{\prime \prime }}_{m-1}})$$

As referenced in the literature [7], this paper has set the parameters for the RTSMFDE method as follows: *N* = 3000, *m* = 2, *c* = 6, *t* = 1, and *s* = 25.

### 2.3. Cosine Pairwise-Constrained Supervised Manifold Mapping

**Figure 2:**
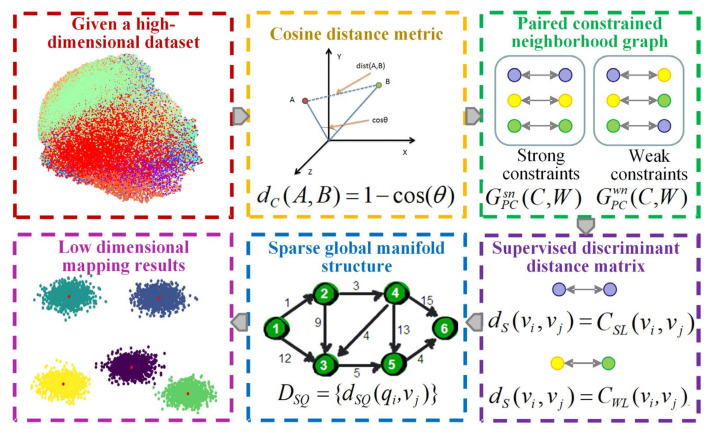
Flowchart of the CPCSMM algorithm.

The utilization of the CPCSMM allows for the reduction of dimensionality in fault features. The process, demonstrated in Figure 2, entails the following specific steps:

(1) Cosine distance measurement

Euclidean distance is typically used in traditional manifold learning methods to measure sample similarity when constructing a neighborhood graph. If the Euclidean distance between two samples is smaller, their similarity is greater. On the contrary, if the Euclidean distance between two samples is larger, their similarity is smaller.

The European distance measurement, however, is not without its shortcomings. Firstly, it is susceptible to the influence of dimensions, resulting in an uncertain range. Secondly, when treating outliers as proximate points, the Euclidean distance metric proves inadequate in accurately depicting the connection between these isolated points and their other nearby neighbors. This inadequacy has the potential to undermine the overall integrity of the neighborhood graph structure.

In comparison to the Euclidean distance measurement, the cosine distance measurement exhibits certain advantages. It mitigates the impact of dimensions and maintains a fixed value range. In addition, the cosine distance measurement shows better robustness when dealing with outliers. Therefore, this chapter uses the cosine distance measurement to measure sample distance in high-dimensional space.

The cosine similarity expression between any two vectors $A=\{A_{i}\}$ and $B=\{B_{i}\}$ is:

(9) $$\cos(\theta )=\frac{A\cdot B}{\left\|A\right\|\left\|B\right\|}=\frac{\sum_{i=1}^{n}A_{i}B_{i}}{\sqrt{\sum_{i=1}^{n}A_{i}^{2}}\sqrt{\sum_{i=1}^{n}B_{i}^{2}}}$$

The cosine distance between vectors *A* and *B* is defined as:

(10) $$d_{C}(A,B)=1-\cos(\theta )$$

It can be observed that the cosine distance ranges from [0, 2].

(2) Pairwising the constrained neighborhood graph

The data set $V=\{v_{i},i=1,\;\;\;\ldots ,n|l(v_{i})\in [1,\;\;\;\ldots ,L]\}$ is provided, where $v_{i}$ represents the sample points, $l(v_{i})$ indicates the label category, and *L* represents the whole number of categories. First, the pairwise constrained neighborhood graph $G_{PC}(C,\;\;W)$ is constructed, where $W(v_{i},\;v_{j})\in \{0,\;\;\;1\}$ represents two constraint types of nearest neighbor points, namely weak constraint and strong constraint. Specifically, the two points belong to the strong constraint type, and the weight is defined as $W(v_{i},\;\;\;v_{j})=1$ if the sample points $v_{i}$ and $v_{j}$ have the same label category. If sample points $v_{i}$ and $v_{j}$ have different label categories, the two points belong to the weak constraint type, and the weight is defined as $W(v_{i},\;\;\;v_{j})=0$. In addition, the constraint set *S_SL_* is constructed for the strong constraint type sample. The constraint set *S_WL_* is constructed for the weak constraint type sample to obtain the paired constraint set:

(11) $$\begin{array}{l} S_{SL}=\{W(v_{i},v_{j})=1,\;l(v_{i})=l(v_{j})\}\\ S_{WL}=\{W(v_{i},v_{j})=0,\;l(v_{i})\neq l(v_{j})\} \end{array}$$

Based on the above definition, a strongly constrained neighborhood graph $G_{PC}^{sn}(C,\;\;W)$ is constructed for the same label class samples. A weakly constrained neighborhood graph $G_{PC}^{wn}(C,\;\;W)$ is constructed for samples of different label categories.

(3) Supervising the discriminant distance matrix

If the constraint relation between any two points $v_{i}$ and $v_{j}$ on the neighborhood graph $G_{PC}^{sn}(C,\;\;W)$ is $W(v_{i},v_{j})\in S_{SL}$, then the distance between them is defined as:

(12) $$C_{SL}(v_{i},v_{j})=\sqrt{1-\exp(\frac{-d_{C}^{2}(v_{i},\;\;v_{j})}{\mu^{\prime }})},\;\;\;W(v_{i},\;\;\;v_{j})\in S_{SL}$$

where $\mu^{\prime }$ is the adjustment coefficient used to curb the overgrowth of inter-class distance and is characterized as the average cosine distance between all the samples.

If the constraint relation between any two points $v_{i}$ and $v_{j}$ on the neighborhood graph $G_{PC}^{wn}(C,\;\;W)$ is $W(v_{i},v_{j})\in S_{WL}$, then the distance between them is defined as:

(13) $$C_{WL}(v_{i},v_{j})=\sqrt{\exp(\frac{d_{C}^{2}(v_{i},\;\;\;v_{j})}{\mu^{\prime }})}-\varphi \;\;,\;\;\;W(v_{i},\;v_{j})\in S_{WL}$$

where φ is the adjustment factor.

The supervised discriminant distance matrix $D_{S}=\{d_{S}(v_{i},v_{j})\}$ of the dataset is constructed based on the above analysis and expressed as follows:

(14) $$d_{S}(v_{i},v_{j})=\left\{\begin{array}{l} C_{SL}(v_{i},v_{j})\;,\;\;W(v_{i},v_{j})\in S_{SL}\\ C_{WL}(v_{i},v_{j})\;,\;\;W(v_{i},v_{j})\in S_{WL} \end{array}\right.$$

(4) Sparse global manifold structure

The distance matrix of a sparse global manifold structure is constructed based on the above theory. The detailed process is as follows.

The manifold topology of the original high-dimensional data set is approximated by randomly selecting some sparse points $Q=\{q_{1},\;\;\;\;q_{2},\;\;\;\cdot \cdot \cdot ,\;\;\;q_{e}\}$ from the data set *V*. Among them, the quantity of sparse points should be less than the total sample number. The global manifold structure matrix $D_{SQ}=\{d_{SQ}(q_{i},v_{j})\}$ among all the sample points and sparse points is constructed. Specifically, the manifold distance before the sparse point $q_{i}$ and the sample point $v_{j}$ is the corresponding supervised discriminant distance if $q_{i}$ is the *k* adjacent point $v_{j}$. Conversely, the approximation of the distance between the two manifolds is achieved by calculating the shortest path between the two points using the Dijkstra algorithm. The corresponding expression is as follows:

(15) $$d_{SQ}(q_{i},v_{j})\;=\left\{\begin{array}{l} d_{S}(q_{i},v_{j})\;\;,\;q_{i}\;\mathrm{is}\;\mathrm{the}\;\mathrm{nearest}\;\mathrm{neighbor}\;\mathrm{of}\;v_{j}\\ \min\{d_{G}(q_{i},v_{j}),d_{G}(q_{i},v_{k})+d_{G}(v_{k},v_{j})\},\;\mathrm{else} \end{array}\right.$$

(5) Low-dimensional mapping results

The low-dimensional mapping result $Y_{Q}$ of a sparse set of points is computed to build a centralized inner product matrix:

(16) $$B_{e}=-\frac{1}{2}H_{e}T_{e}H_{e}$$

where $T_{e}$ represents the square matrix of sparse point manifold distance matrix ${D^{\prime }}_{SQ}=\{d_{SQ}(q_{i},q_{j})\}$ and $H_{e}$ represents a centralized matrix.

The maximum *d* eigenvalues $B_{e}$ are calculated, where *d* represents the intrinsic dimension. The *i*-th eigenvalue and its corresponding eigenvector are represented by parameters $\lambda_{i}$ and $\zeta_{i}$, respectively. Then, the DR result of the sparse point set is:

(17) $$Y_{Q}=\left[\begin{array}{l} \sqrt{\lambda_{1}}\cdot \zeta_{1}^{T}\\ \sqrt{\lambda_{2}}\cdot \zeta_{2}^{T}\\ \;\;\;\;\;\vdots \\ \sqrt{\lambda_{d}}\cdot \zeta_{d}^{T} \end{array}\right]$$

The low-dimensional mapping result $Y_{G}$ of other sample points (not sparse points) is calculated as:

(18) $$Y_{G}=\frac{1}{2}{Y\prime }_{Q}({\bar{T}}_{a}-T_{a})$$

where ${\bar{T}}_{a}$ represents the column average matrix of $T_{a}$, and $T_{a}$ represents the square matrix of $D_{SQ}$. ${Y^{\prime }}_{Q}$ can be expressed as follows:

(19) $${Y^{\prime }}_{Q}=\left[\begin{array}{l} \zeta_{1}^{T}/\sqrt{\lambda_{1}}\\ \zeta_{2}^{T}/\sqrt{\lambda_{2}}\\ \;\;\;\;\;\vdots \\ \zeta_{d}^{T}/\sqrt{\lambda_{d}} \end{array}\right]$$

### 2.4. Adaptive Chaotic Aquila Optimization Support Vector Machine

**Figure 3:**
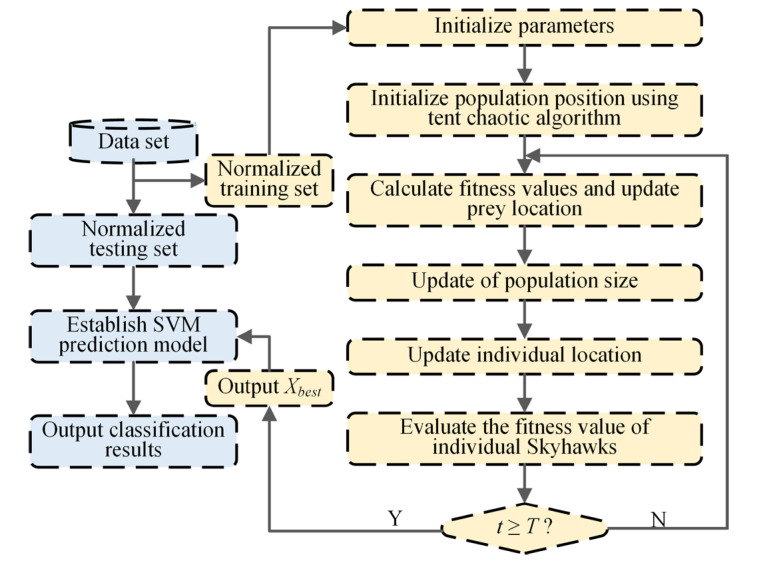
Flowchart of the ACAO-SVM method.

The optimization process of key parameters of the SVM classifier applies the adaptive chaotic Aquila optimization (ACAO) method. Moreover, an intelligent fault classification method of ACAO-SVM is proposed. The process, depicted in Figure 3, is detailed through the following specific steps:

(1) Data preprocessing. The training and test sets are created by randomly dividing the input feature set. In addition, (v−min)/(max−min) is used to normalize the training and test sets to [0, 1]. $v^{\prime }$ and *v* represents the normalized eigenvalues and the original, and min and max represent the minimum and maximum eigenvalues;
(2) Initializing the ACAO method parameters. The minimum population size *P_min_* is 5, the maximum population size *P_max_* is 30, the maximum number of initial iterations *T* is 200, the upper limit UB of the optimization problem is [0.001, 0.001], the lower limit LB of the optimization problem is [100, 100], and the individual position of the Aquila is ($c^{\prime },\;\;\;g$);
(3) The population location is initialized by the tent chaotic mapping method (details shown in Equations (20) and (21)). In the *D_im_*-dimension space, generates the tent chaotic sequence $Z=\{z_{i}\}$ with different trajectories:

(20) $$z_{i+1}=\left\{\begin{array}{l} 2\times z_{i},\;\;\;\;\;\;\;\;\;\;\;\;\;0\leq z_{i}\leq 1/2\\ 2\times (1-z_{i}),\;\;\;\;1/2<z_{i}<1 \end{array}\right.$$

*Z* carrier to the solution space is used to generate the initial individual space position X(1):

(21) $$X(1)=L{\;}_{B}+(U_{B}-L{\;}_{B})\times Z$$

(4) Adaptive updating of Aquila’s population size. Aquila’s population size is adaptively updated using the linear reduction method (Equation (22)). The computational complexity of the Aquila optimizer (AO) is determined by the maximum number of iterations *T*, the optimization solution dimension *D_im_*, and the population size *P*. Thus, to enhance the operational efficiency of the AO, the original constant population size strategy is replaced with a linear reduction adaptive population size update method;

(22) $$P(t+1)=round\;\;\;\left[\frac{\left(P_{\min}-P_{\max}\right)}{T}\times t+P_{\max}\right]$$

where *t* represents the current iteration. $P_{\min}$ and $P_{\max}$ represent the minimum and the maximum population size, respectively, and round [ ⋅ ] represents the integer function.

(5) The fitness value of each Aquila individual is calculated in the current iteration. The fitness value is defined as the average error classification rate after conducting a three-fold cross-validation on the training set. To achieve this, the training set is normalized and divided into three groups. One group is randomly selected as the sub-validation set, while the remaining two groups are treated as the training set, resulting in the creation of three models. The fitness value is obtained by calculating the average error classification rate of each model on its corresponding validation set. The current target prey position is identified as the position $X_{\mathrm{best}}$ of the Aquila individual with the lowest fitness value in the current iteration. Consequently, the optimization process of the SVM classifier parameters aims to discover the global minimum fitness value;
(6) Updating the individual position of the Aquila. The strategies include soaring at high altitudes with vertical dives, glide attacks at close range, contour flying, slow descent attacks at low altitudes, and stalking and capturing prey, as detailed in Equations (23)–(26):

(23) $$\begin{array}{l} X(t+1)=X_{\mathrm{best}}(t)\times (1-\frac{t}{T})+[X_{M}(t)-X_{\mathrm{best}}(t)\times rand]\\ X_{M}(t)=\frac{1}{P}\sum_{i=1}^{P}X_{i}(t) \end{array}$$

where *rand* represents the random number between [0, 1], $X_{\mathrm{best}}(t)$ represents the best individual position under *t* iteration (i.e., the prey position), and $X_{M}(t)$ denotes the mean position of all individuals in the current iteration.

The Aquila hovers over its prey, preparing to land and launch an attack. This process is called contour flight for a short glide attack and can be mathematically expressed as follows:

(24) $$\begin{array}{l} X(t+1)=X_{\mathrm{best}}(t)\times Levy(D)+X(t)+(y-x)\times rand\\ \;x=r^{\prime }\times \sin(\theta ),\;\;y=r^{\prime }\times \cos(\theta )\\ \;\;r^{\prime }=r_{1}+0.00565\times D_{1},\;\;\theta =-0.005\times D_{1}+3\pi /2\\ Levy(D)=0.01\times \frac{u\times \sigma }{|v{|}^{2/3}},\;\;\sigma =\frac{\Gamma (2.5)\times \sin(1.5\pi /2)}{\Gamma (1.25)\times 1.5\times 2^{0.25}} \end{array}$$

where *u* and *v* are random numbers between [0, 1], $D_{1}$ is an integer from 1 to *D_im_*, and $r_{1}$ is the number of cycles between 1 and 20.

The Aquila bird hovers over its prey, poised to descend and initiate an attack. This maneuver, known as contour flight, involves a brief gliding descent and can be expressed mathematically as follows:

(25) $$X(t+1)=0.1\times [X_{\mathrm{best}}(t)-X_{M}(t)]-rand+0.1\times [(U_{B}\;-\;L_{B})\times rand+L{\;}_{B}]$$

According to the random target movement, the Aquila walks on the ground to attack and capture the prey. The corresponding mathematical expression is as follows:

(26) $$\begin{array}{l} X(t+1)=F_{Q}(t)\times X_{\mathrm{best}}(t)-G_{1}\times X(t)\times rand-G_{2}\times Levy(D)+G_{1}\times rand\\ F_{Q}(t)=t^{(2\times rand-1)/{\left(1-T\right)}^{2}},\;\;\;\;\;G_{1}=2\times rand-1,\;\;\;\;\;G_{2}=2\times (1-t/T) \end{array}$$

where $F_{Q}(t)$ represents the mass function under *t* iteration. $G_{1}$ is the moving parameter. $G_{2}$ is the flight slope.

(7) Evaluating the position of individual Aquilas and the prey. In the present iteration, if either the fitness values of the individual or the prey surpass their historical values, the original positions of the individual or the prey should be replaced with the updated positions. Alternatively, if the historical positions of either the individual or the prey are superior in terms of fitness values, these historical positions are retained;
(8) Determining whether the iteration is terminated. If the maximum number of iterations is reached, the entire cycle is halted. Otherwise, steps (4)–(7) are iteratively repeated until the specified condition is satisfied;
(9) Determining the final prey location. At the termination of the iteration, the final captured prey position is determined by outputting the location $X_{\mathrm{best}}$ of the best individual in the Aquila population;
(10) Establishing the SVM prediction model. The SVM prediction model is established according to the parameter optimization result $X_{\mathrm{best}}$;
(11) Sample classification. The normalized test set is fed into the SVM prediction model for intelligent classification, which then generates the predicted fault type for the test samples.

## 3. Experimental Validation

### 3.1. Self-Priming Centrifugal Pump Experiment Platform and Data Collection

**Figure 4:**
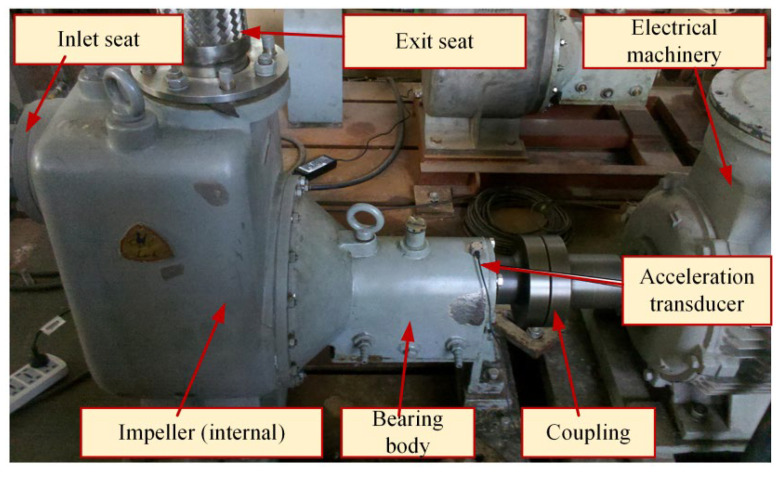
The experimental platform of the self-priming centrifugal pump.

**Figure 5:**
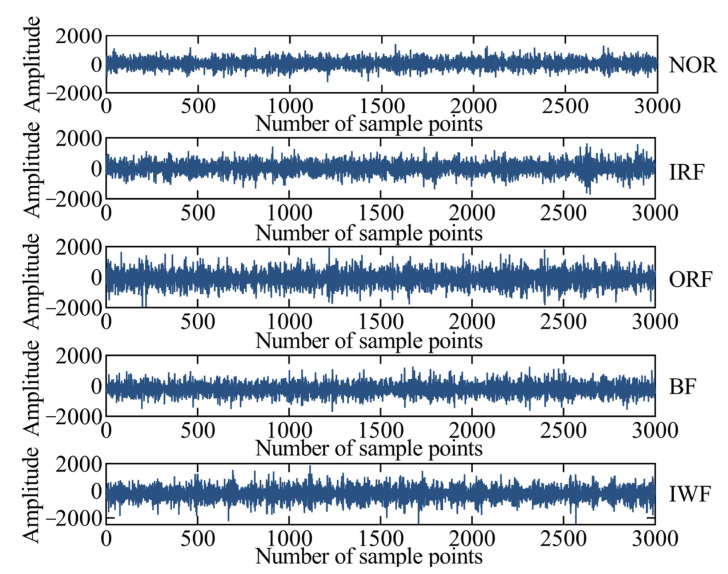
The time-domain waveforms of the self-priming centrifugal pump.

The experimental platform of a self-priming centrifugal pump is shown in Figure 4 and mainly comprises a motor, coupling, bearing body, impeller, inlet seat, and outlet seat [32]. The vibration acceleration signals of the self-priming centrifugal pump in five states are collected using an acceleration sensor, data acquisition instrument, and computer under the condition of rotation speed of 2900 r/min and sampling frequency of $f_{s}$ = 10,240 Hz. Moreover, the time-domain waveforms corresponding to the normal (NOR), inner ring fault (IRF), outer ring fault (ORF), ball fault (BF), and impeller wear fault (IWF) are also collected and shown in Figure 5. Table 1 provides a description of the experimental data pertaining to the fault diagnosis.

### 3.2. Fault Diagnosis Results and Comparative Analysis

#### 3.2.1. Fault Diagnosis Results

**Figure 6:**
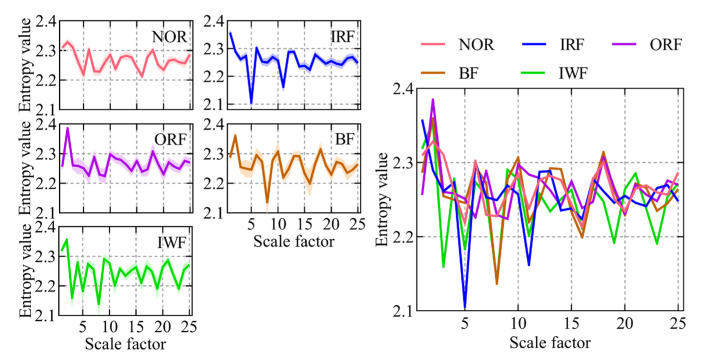
The RTSMFDE entropy curves of the self-priming centrifugal pump under five states.

A case study involving a self-priming centrifugal pump was carried out to scrutinize the proposed intelligent fault diagnosis method tailored for such pumps. First, the RTSMFDE fault feature extraction method defined in Equations (1)–(8) was used to extract the fault feature information for the entropy domain of 450 groups of signals under five states of a self-priming centrifugal pump. The corresponding entropy curve is shown in Figure 6.

**Figure 7:**
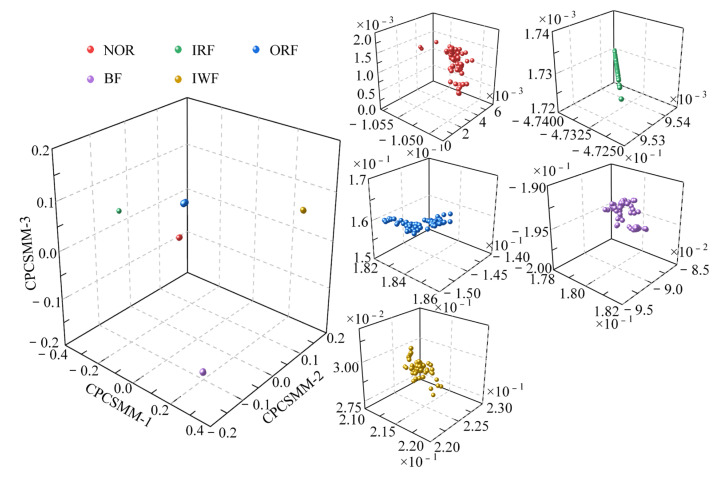
DR results obtained by the CPCSMM method.

In most scale factors, the entropy values of the five states of the self-priming centrifugal pump, as indicated by Figure 6, exhibited significant differences; this validates that the proposed RTSMFDE method can effectively extract characteristic information from the signal, thereby facilitating easy distinction of fault types in the self-priming centrifugal pump. However, the RTSMFDE entropy values of different states were relatively close in some scale factors. For example, when the scale factor is 6, it can be difficult to distinguish the RTSMFDE entropy of IRF and NOR samples. Similarly, when the scale factor is 2, the RTSMFDE entropy values of IWF and BF samples are not easily distinguishable. In light of this, the utilization of the CPCSMM becomes essential for reducing the dimensionality of the RTSMFDE set. The entropy-manifold features obtained according to Equations (9)–(19) are shown in Figure 7. The parameters of the CPCSMM scheme were determined by cross-validation, i.e., the intrinsic dimension was 3, the nearest neighbor parameter was 81, and the adjustment coefficient φ was 0.45.

As illustrated in Figure 7, the samples representing the five states of the self-priming centrifugal pump were distinctly segregated within the CPCSMM mapping space. Notably, there is no overlap or blurring of boundaries between samples belonging to different categories. In addition, the sample aggregation of the same category is improved. According to the results, the proposed method successfully identifies and extracts sensitive fault features from a high-dimensional feature set. Moreover, it effectively distinguishes between the different fault types of the self-priming centrifugal pump in the mapping space.

**Figure 8:**
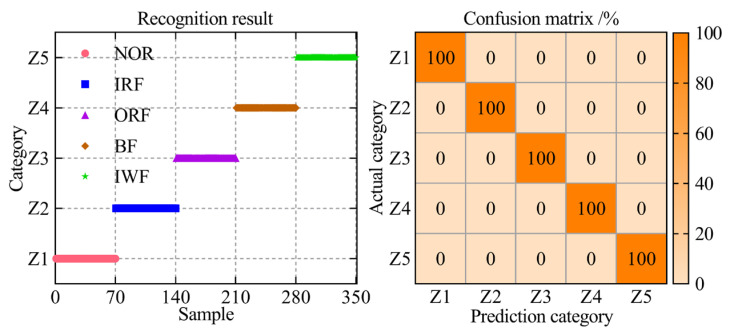
The fault diagnosis results of the proposed method.

In the ultimate step towards enabling intelligent fault diagnosis for a self-priming centrifugal pump, the entropy-manifold feature set extracted through the RTSMFDE+CPCSMM was employed as input data for the ACAO-SVM classifier, facilitating precise and accurate fault classification. The number of training and test samples is shown in Table 1. The key parameters of the support vector machine model were optimized using the adaptive chaotic Aquila optimization method. The optimization results were ${c^{\prime }}_{\mathrm{best}}$ = 27.74 and $g_{\mathrm{best}}$ = 55.48. Then, the ACAO-SVM prediction model was established according to the optimization results, and the test set of the self-priming centrifugal pump was fed into the prediction model to accomplish intelligent fault classification. The identification results and the confusion matrix are shown in Figure 8.

According to Figure 8, the ACAO-SVM classifier can accurately identify the fault types of 350 test samples of the self-priming centrifugal pump with a recognition rate of 100%. The analysis conclusively demonstrated that the proposed intelligent fault diagnosis method, incorporating the RTSMFDE+CPCSMM features, excels at accurately diagnosing the fault types in the self-priming centrifugal pump.

#### 3.2.2. Comparative Experiments of Different Fault Feature Extraction Methods

**Figure 9:**
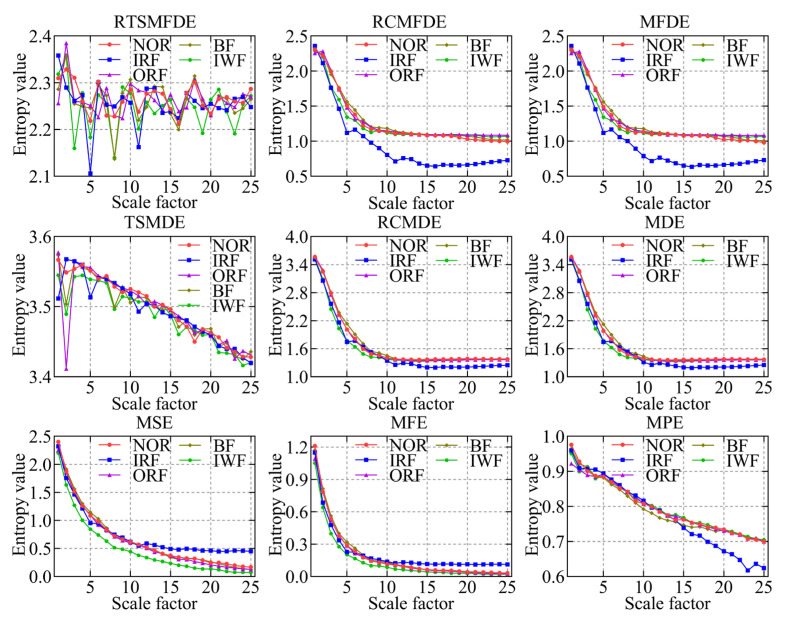
Mean entropy curves using nine feature extraction methods.

**Figure 10:**
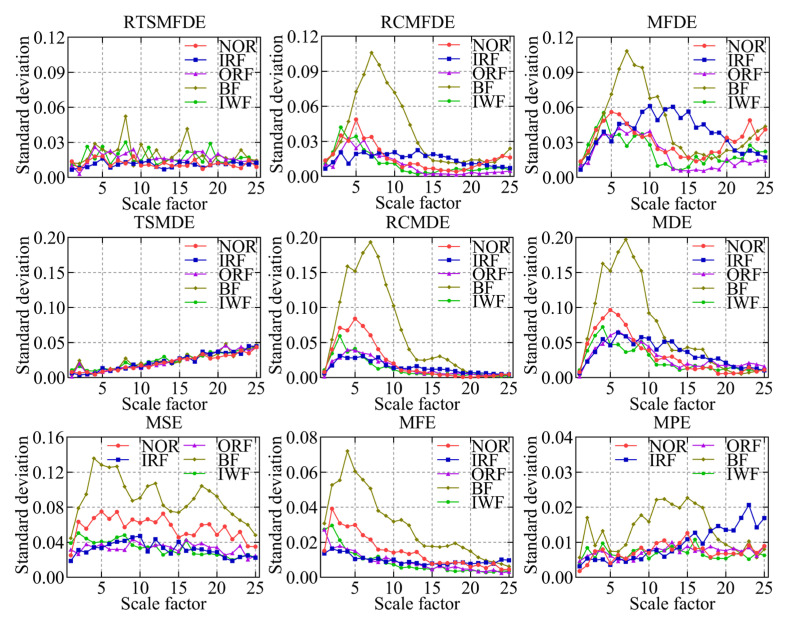
The standard deviation of entropy values for nine feature extraction methods.

To validate the effectiveness of the RTSMFDE method, a comparison was conducted with several existing feature extraction methods. These methods included multiscale permutation entropy (MPE) [33], multiscale fuzzy entropy (MFE) [34], multiscale sample entropy (MSE), MDE [35], refined composite multiscale dispersion entropy (RCMDE) [36], time-shift multiscale dispersion entropy (TSMDE) [37], MFDE [38], and refined composite multiscale fluctuation dispersion entropy (RCMFDE) [13]. Table 2 presents the parameter settings for each method. Figure 9 and Figure 10 show the mean entropy curve and standard difference of fault signals extracted by nine feature extraction methods for the self-priming centrifugal pump, respectively.

According to Figure 9 and Figure 10, the following conclusions can be drawn.

IRF and IWF can be distinguished in the MDE, MSE, MFE, and MPE analysis results. However, the mean entropy curves of the remaining three samples were relatively close. Compared with the above methods, MFDE analysis results showed better differentiation of the self-priming centrifugal pump faults for some scale factors. The above analysis shows that MFDE feature extraction was superior to MDE, MSE, MFE, and MPE.

In addition, the mean entropy curves obtained by MFDE and RCMFDE (or MDE and RCMDE) for the same signal analysis were roughly the same. However, compared with the MFDE method (or MDE method), the RCMFDE method (or RCMDE method) had a lower standard entropy deviation on most scale factors. This is because the RCMFDE and RCMDE methods adopt a complex multiscale decomposition and refined operation; therefore, they can obtain more stable entropy domain features.

Upon comparison with RCMFDE (or RCMDE), it becomes evident that the RTSMFDE (or TSMDE) exhibited an enhanced discriminatory capacity across various self-priming centrifugal pumps. Additionally, it was observed to have a lower standard deviation for most scale factors. This observation shows that the time-shifted multiscale decomposition can mine more accurate fault features in the entropy domain than the composite multiscale decomposition.

Finally, when compared with the eight fault feature extraction methods, the RTSMFDE analysis demonstrated a distinct and significantly differentiated change in the mean entropy curve for each operational state. This distinctive trend was instrumental in distinguishing various fault types within self-priming centrifugal pumps, thereby affirming the superiority of this feature extraction method.

**Figure 11:**
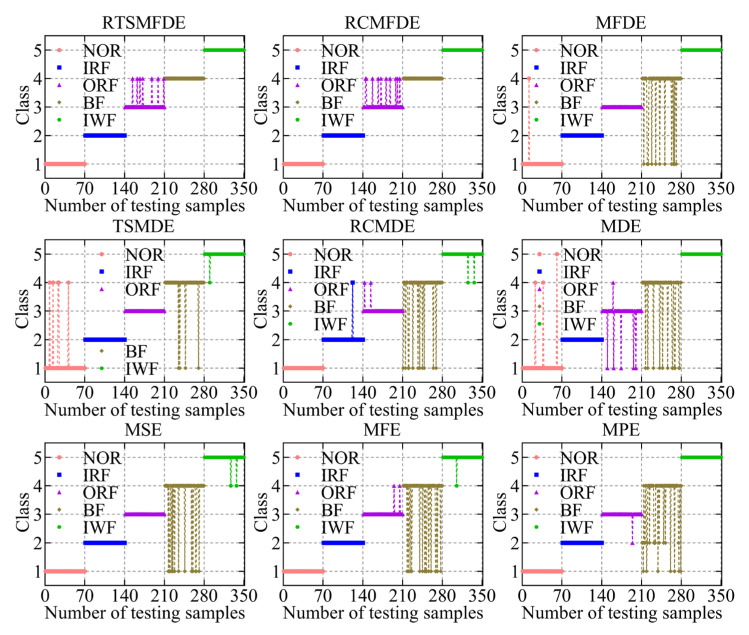
Recognition results of nine feature sets by the ACAO-SVM classifier.

**Figure 12:**
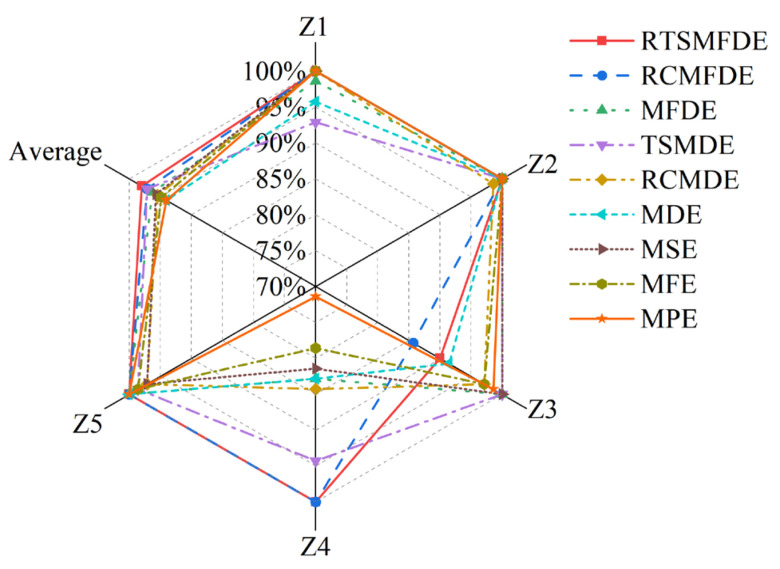
Accuracy recognition rates of nine feature sets.

The fault feature sets obtained from the aforementioned nine methods were utilized as inputs for the ACAO-SVM classifier, enabling intelligent fault identification. The classification results and correct recognition rate are shown in Figure 11 and Figure 12, respectively. The following conclusions can be drawn.

First, compared with MSE (15 wrong classifications), MFE (18 wrong classifications), MPE (21 wrong classifications), and MDE (21 wrong classifications), MFDE (13 wrong classifications) had fewer wrong categories. Thus, the effectiveness of the MFDE method in fault feature extraction of self-priming centrifugal pumps was verified.

Compared with MFDE, RCMFDE (10 wrong classifications) had three more correct identifiers. Similarly, compared with MDE, RCMDE (16 wrong classifications) had five more correct identifiers. This enhancement can be attributed to the utilization of composite multiscale decomposition and refined methods within RCMFDE and RCMDE. These techniques lead to more stable feature extraction outcomes and contribute to improved effectiveness in subsequent intelligent fault identification.

Compared with the RCMFDE method that employs composite multiscale decomposition, the RTSMFDE method using time-lapse multiscale decomposition (seven wrong classifications) reduced the number of wrong classifications of the self-priming centrifugal pump by three. Similarly, compared with the RCMDE method using the composite multiscale decomposition, the TSMDE method using the time-shifted multiscale decomposition (10 wrong classifications) reduced the number of wrong classifications of the self-priming centrifugal pump by 6. This is because the time-shift multiscale decomposition directly constructs the coarse-grained sequence from the original signal to avoid dynamic mutation deficiency of the original signal caused by the complex multiscale decomposition. Consequently, the feature information that can easily distinguish fault types can be extracted.

Finally, the average correct recognition rate of the final RTSMFDE feature set (98.00%) was higher than that of the RCMFDE method by 0.86%, higher than the MFDE method by 1.71%, higher than the TSMDE method by 0.86%, higher than the RCMDE method by 2.57%, higher than the MDE method by 4.00%, higher than the MSE method by 2.29%, higher than the MFE method by 3.14%, and higher than the MPE method by 4.00%. In conclusion, the ability of the RTSMFDE method to extract fault characteristic information of a self-priming centrifugal pump was proven. The extraction effect is better than eight algorithms for fault feature extraction.

#### 3.2.3. Comparative Experiments of Different DR Methods

The CPCSMM method was compared with LLE [19], Isomap [39], LTSA [21], linear discriminant analysis (LDA) [16], weighted isometric mapping (WIso) [40], and pairwise-constrained supervised manifold mapping (PCSMM) to verify its advantages. Visualization, DR performance index, and the correct recognition rate of the three aspects are indicators for comprehensive evaluation.

(1) Visual comparison experiment

**Figure 13:**
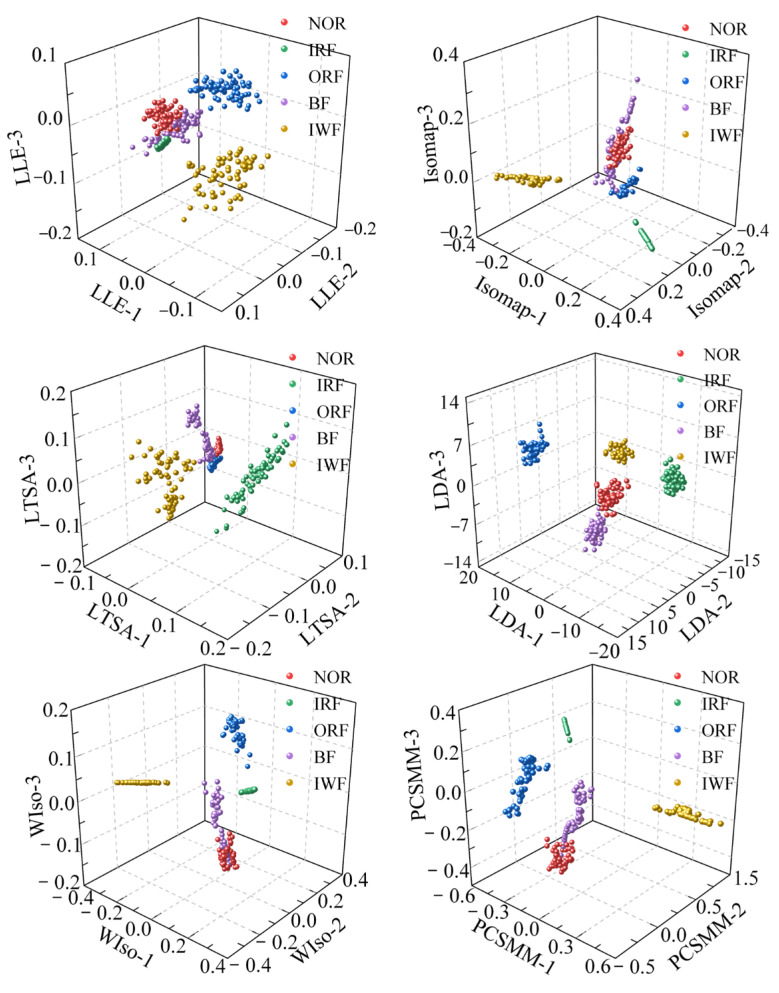
Visualization results using different DR methods.

To reduce the dimensionality of the RTSMFDE feature set, six comparative DR methods were utilized. The visualization outcomes, depicted in Figure 13, offer insights into the effectiveness of these methods. Additionally, Table 3 provides the parameters of each DR method, carefully determined through cross-verification.

According to Figure 7 and Figure 13, the sample aggregation of each state of the self-priming centrifugal pump was poor according to the visual results of the unsupervised DR method, accompanied by a serious aliasing phenomenon. Based on the visualized outcomes of the supervised DR method, it becomes evident that while the fault types within the self-priming centrifugal pump are nearly distinguishable, there was a relatively weak clustering effect among samples of the same operational state. The boundaries of some heterogeneous samples are not obvious, such as the boundaries of the NOR and BF samples in the visualized results of the LDA, WIso, and PCSMM. Furthermore, some samples are relatively far from the clustering center. Compared with the above method, the CPCSMM visualization results showed that the sample aggregation of each state was the best and that the fault types of the self-priming centrifugal pump could be distinguished. The most favorable visualization outcomes were yielded by the CPCSMM method, as indicated by the analysis results presented above.

(2) Comparison experiment of DR performance index

To quantify the DR effect of the aforementioned method, the ratio of inter-class divergence to intra-class divergence was defined as the performance index. A higher ratio signifies a greater concentration of samples from the same class within the mapping space. Simultaneously, it implies that samples from different classes are more widely dispersed, indicating superior DR performance. The statistical results of the performance indices for the seven methods are shown in Table 4.

According to Table 4, the following conclusions can be drawn.

The DR performance indexes of the supervised DR methods (such as LDA, WIso, PCSMM, and CPCSMM) were significantly higher than those of unsupervised DR methods (such as LLE, Isomap, and LTSA), indicating that DR guided by sample label information can improve the DR performance.

Secondly, in the supervised DR method based on Euclidean distance, the DR performance index of the PCSMM method was 3.28 × 10^1^, which was 7.40 and 3.00 higher than LDA and WIso, respectively. The LDA method, being primarily a linear DR method, is not well-suited for effectively handling the nonlinear fault feature sets found in self-priming centrifugal pumps. Although the WIso method is a nonlinear DR method, it distorts the manifold structure of an input feature set. Furthermore, the DR performance of this method is intricately linked to the weight factor parameters. In contrast, the PCSMM method combines the benefits of pair-constrained neighborhood graphs, supervised learning, and sparse global manifold structures. Consequently, it showcases superior DR performance metrics.

Finally, the DR performance of the CPCSMM method was improved by three orders of magnitude compared to the PCSMM. This improvement is attributed to the fact that the Euclidean distance in the PCSMM method fails to accurately depict the relationship between outliers and other samples. In contrast, the CPCSMM method utilizes sample relation for cosine distance measurement, which is more appropriate for handling outliers. Consequently, the CPCSMM method yields precise DR results. Based on the above analysis, it is evident that the CPCSMM method outperforms other methods in terms of the DR performance index.

(3) Comparison experiment of correct recognition rate

**Figure 14:**
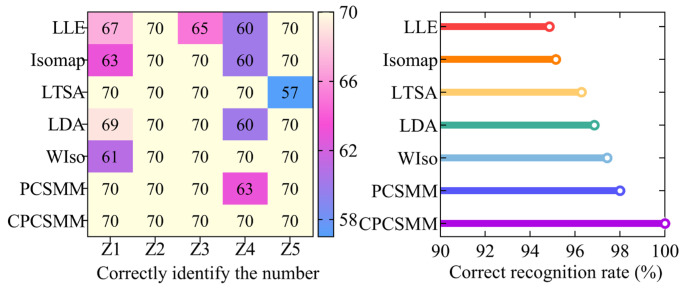
Recognition results of different DR methods.

The ACAO-SVM classifier executed the task of intelligent fault classification using the seven low-dimensional features. Figure 14 illustrates the recognition results and the corresponding correct recognition rate. Table 1 provides information regarding the number of training and test samples.

Figure 14 reveals the classification errors for different methods, namely LLE, Isomap, LTSA, LDA, WIso, and PCSMM, with 18, 17, 13, 11, 9, and 7 test samples, respectively, being wrongly classified. Remarkably, the CPCSMM method achieved perfect accuracy, correctly classifying all test samples. Moreover, the correct recognition rate of the CPCSMM method reached 100%, surpassing that of LLE, Isomap, LTSA, LDA, WIso, and PCSMM by 5.14%, 4.86%, 3.71%, 3.14%, 2.57%, and 2.00%, respectively. Based on this analysis, it is irrefutable that the CPCSMM method stands out as having the highest correct recognition rate when compared to the other methods.

In summary, the CPCSMM method distinguishes itself from unsupervised DR methods like LLE, Isomap, or LTSA, as well as supervised DR methods such as LDA, WIso, and PCSMM. The CPCSMM method stands out with its superior visualization effect, exceptional DR index, and highest correct recognition rate. These results confirm the method’s superiority in reducing the dimensionality of the fault feature set in self-priming centrifugal pumps.

#### 3.2.4. Comparative Experiments of Different Intelligent Fault Diagnosis Methods

**Figure 15:**
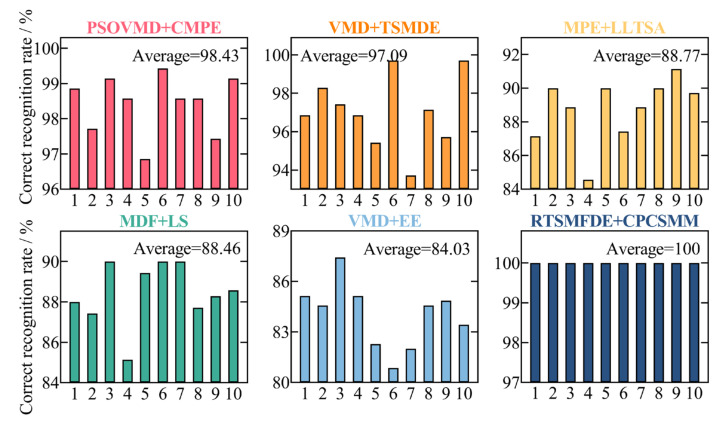
Recognition results of ten independent experiments with different models.

To assess the accuracy of the proposed intelligent fault diagnosis method tailored for self-priming centrifugal pumps, which leverages the entropy-manifold features of RTSMFDE and CPCSMM, a comparative analysis was conducted. This involved a comparison between the proposed method and five existing intelligent fault diagnosis methods. Model 1 consisted of particle swarm optimization-based variational mode decomposition (PSO-VMD), composite multiscale permutation entropy (CMPE), and reverse cognitive fruit fly optimization algorithm–extreme learning machine [41]. Model 2 comprised variational mode decomposition (VMD), TSMDE, and the vibration Harris Aquila optimization support vector [37]. Model 3 comprised MPE, linear local tangent space alignment (LLTSA), and a least square support vector machine [42]. Model 4 consisted of multi-domain features (MDF), Laplace score (LS), and the particle swarm optimization support vector machine [43]. Model 5 comprised VMD, energy entropy (EE), and SVM [44]. Figure 15 shows the diagnostic results of 10 independent experiments on self-priming centrifugal pump data by comparing five fault diagnosis methods and the proposed fault diagnosis method (defined as Model 6). Table 5 lists the parameters of each fault diagnosis method based on related literature.

Figure 15 illustrates the performance of the proposed intelligent fault diagnosis method utilizing the entropy-manifold feature of RTSMFDE+CPCSMM. Notably, this method achieved the highest average recognition rate (100%). The observed rates were significantly higher, with improvements of 1.57%, 2.91%, 11.23%, 11.54%, and 15.97% compared to Models 1–5, respectively.

The causal factors contributing to these phenomena are as follows:

This outcome was attainable due to the redundancy of information within the fault feature sets extracted by Models 1 and 2. This redundancy had a detrimental impact on the classification accuracy of the subsequent classifiers. In Model 3, despite the use of the LLTSA method for feature dimensionality reduction, the MPE method neglects the amplitude information within the original sequence. Furthermore, MPE solely takes into account a single coarse-grained sequence at the same scale, thereby overlooking potentially valuable information from other sources. Similarly, within the framework of Model 4, the LS method is employed for feature selection. Nevertheless, the utilization of the MDF method for extracting fault features from multiple domains results in time-consuming processes and an excess of redundancies within the feature set. Within Model 5, only a limited set of four fault features is extracted from each signal, resulting in an incomplete exploration of the essential fault information associated with the self-priming centrifugal pump.

In a direct comparison with existing intelligent fault diagnosis methods, the proposed methodology adeptly amalgamates the unique advantages offered by RTSMFDE in fault feature extraction, CPCSMM in fault feature dimensionality reduction, and ACAO-SVM in intelligent fault classification. As a consequence, the proposed method achieves the highest recognition accuracy rate. This comprehensive analysis unequivocally establishes that the intelligent fault diagnosis method, incorporating the RTSMFDE+CPCSMM entropy-manifold features, is markedly more well-suited for the precise diagnosis of critical components within self-priming centrifugal pumps.

## 4. Conclusions

This paper proposed an intelligent fault diagnosis method for self-priming centrifugal pumps. The method integrates the advantages of RTSMFDE in fault information mining, CPCSMM in sensitive feature extraction, and ACAO-SVM in fault identification. The fault diagnosis analysis for a self-priming centrifugal pump has demonstrated the effectiveness and accuracy of the proposed method in identifying fault types. Based on these analyses, the following conclusions can be drawn:

(1) With a recognition rate of 100%, the proposed intelligent fault diagnosis methods, RTSMFDE, CPCSMM, and ACAO-SVM, accurately identify the fault type for self-priming centrifugal pumps. Furthermore, the average correct recognition rate of the proposed methods surpasses that of the existing five intelligent fault diagnosis methods by up to 15.97%;
(2) The fault feature information of self-priming centrifugal pumps can be effectively extracted by the RTSMFDE method. When compared to eight existing fault feature extraction methods, namely MPE, MFE, MSE, MDE, RCMDE, TSMDE, MFDE, and RCMFDE, the correct recognition rate of the RTSMFDE can be improved by up to 4.00%;
(3) The CPCSMM method has the optimal visualization effect, the optimal DR performance index, and the highest correct recognition rate compared to the six DR methods (LLE, Isomap, LTSA, LDA, WIso, and PCSMM).

In the future, the authors intend to apply the proposed fault diagnosis method to identify and analyze additional faults in self-priming centrifugal pumps.

## Figures and Tables

**Table 1:** The experimental description of the self-priming centrifugal pump under different fault states.

Status	Tag	Number of Training Samples	Number of Testing Samples	Sample Count
Normal (NOR)	Z1	20	70	90
Inner-ring fault (IRF)	Z2	20	70	90
Outer-ring fault (ORF)	Z3	20	70	90
Bearing fault (BF)	Z4	20	70	90
Impeller wear fault (IWF)	Z5	20	70	90

**Table 2:** Parameter settings for different methods.

Entropy Method	Parameter Setting
MPE	*N* = 3000, *m* = 6, *t* = 1, *s* = 25
MFE	*N* = 3000, *m* = 2, *r* = 0.15*SD*, *n* = 2, *t* = 1, *s* = 25
MSE	*N* = 3000, *m* = 2, *r* = 0.15*SD*, *t* = 1, *s* = 25
MDE	*N* = 3000, *m* = 2, *c* = 6, *t* = 1, *s* = 25
RCMDE	
TSMDE	
MFDE	
RCMFDE	
RTSMFDE	

**Table 3:** Parameter settings for different DR methods.

DR Method	Type	Parameter Setting
LLE	Unsupervised	*d* = 3, *K* = 81
Isomap	Unsupervised	*d* = 3, *K* = 52
LTSA	Unsupervised	*d* = 3, *K* = 91
LDA	Supervised	*d* = 3
WIso	Supervised	*d* = 3, *K* = 49, *w* = 0.76
PCSMM (Euclidean distance)	Supervised	*d* = 3, *K* = 55, φ = 0.55
CPCSMM (cosine distance)	Supervised	*d* = 3, *K* = 81, φ = 0.45

**Table 4:** The DR performance metrics for different methods.

DR Method	DR Performance Indicators			DR Time(s)
	Divergence between Classes	Intra-Class Divergence	Ratio	
LLE	5.03 × 10^−3^	1.63 × 10^−3^	3.09	0.47
Isomap	6.76 × 10^−2^	4.20 × 10^−3^	1.61 × 10^1^	1.94
LTSA	4.60 × 10^−3^	2.06 × 10^−3^	2.23	0.72
LDA	1.65 × 10^2^	6.50	2.54 × 10^1^	0.04
WIso	5.51 × 10^−2^	1.85 × 10^−3^	2.98 × 10^1^	1.78
PCSMM	2.58 × 10^−1^	7.87 × 10^−3^	3.28 × 10^1^	0.52
CPCSMM	9.85 × 10^−2^	1.81 × 10^−6^	5.44 × 10^4^	0.72

**Table 5:** Parameter settings of different fault diagnosis methods.

Model	Feature Extraction	Parameter Setting
Model 1	PSOVMD+CMPE	*P* = 50, *T* = 100, *c*_1_ = *c*_2_ = 2, *m* = 6, λ=1, *s* = 12
Model 2	VMD+TSMDE	Decomposition number = 4, *a* = 2000, *m* = 3, τ=1, *c* = 6, *s* = 20
Model 3	MPE+LLTSA	*m* = 4, τ=1, *s* = 12, *d* = 3, *K* = 27
Model 4	MDF+LS	Decomposition number = 4, *a* = 2000, *m* = 3, λ=1
Model 5	VMD+EE	Decomposition number = 4, *a* = 2000
Model 6	RTSMFDE+CPCSMM	*c* = 6, *m* = 2, *t* = 1, *s* = 25, *d* = 3, *K* = 81, φ = 0.45

## Data Availability

Not applicable.

## Abbreviations

AO	Aquila optimizer
ACAO	adaptive chaotic Aquila optimization
ACAO-SVM	adaptive chaotic Aquila optimization support vector machine
BF	ball fault
CPCSMM	cosine pairwise-constrained supervised manifold mapping
CMPE	composite multiscale permutation entropy
DR	dimensionality reduction
EE	energy entropy
IRF	inner ting fault
Isomap	isometric mapping
IWF	impeller wear fault
LE	Laplacian Eigenmaps
LDA	linear discriminant analysis
LLE	locally linear embedding
LTSA	local tangent space alignment
LLTSA	linear local tangent space alignment
LS	Laplace score
MDE	multiscale dispersion entropy
MDF	multi-domain features
MFDE	multiscale fluctuation dispersion entropy
MFE	multiscale fuzzy entropy
MPE	multiscale permutation entropy
MSE	multiscale sample entropy
NOR	normal
ORF	outer ring fault
PCSMM	pairwise-constrained supervised manifold mapping
PSO-VMD	particle swarm optimization-based variational mode decomposition
RCMDE	refined composite multiscale dispersion entropy
RCMFDE	refined composite multiscale fluctuation dispersion entropy
RTSMFDE	refined time-shift multiscale fluctuation dispersion entropy
SVM	support vector machine
TSMDE	time-shift multiscale dispersion entropy
VMD	variational mode decomposition
WIso	weighted isometric mapping
